# The Question of Lag: An Exploration of the Relationship Between Conductor Gesture and Sonic Response in Instrumental Ensembles

**DOI:** 10.3389/fpsyg.2020.573030

**Published:** 2020-12-10

**Authors:** Cory D. Meals

**Affiliations:** Moores School of Music, University of Houston, Houston, TX, United States

**Keywords:** conducting, musical coordination, ensemble performance, entrainment, music

## Abstract

Group musical performance, especially large instrumental ensembles, present the outward appearance of an asymmetric, temporally immediate stimulus-response relationship between conductor and ensemble. Interestingly, anecdotal reports from both conductors and performers indicate a degree of variability in the timing of orchestral response to the conductor’s gestures. This observation is not present in anecdotal accounts of other instrumental ensemble settings, like wind bands, but commonplace occurrence among orchestral musicians indicates the potential presence of greater complexity in the observed relationship. This study investigates both the quality and quantity of temporal lag between conductor and ensemble in two common instrumental ensemble configurations – wind bands and orchestras – in an effort to describe the interplay present within conducted group performance. The findings indicate that the anecdotally identified lag is present within all ensemble types, and that it presents a flexible, dynamic temporal relationship between conductor and ensemble. Additionally, both the quantity and quality of lag values are significantly different between ensemble types, experience levels, and musical content. Several avenues for future research are identified, and confounds within the sampled ensembles are examined for their potential roles in the observed relationships.

## Introduction

The lights of the hall dim and the musicians, who had until recently been going through final performance preparations, fall silent. No sooner has the last sound dissipated than the stage door opens and the conductor emerges. As they stride confidently through the ensemble, musicians rise in unison and the audience recognizes all with warm applause.

From aside their podium the conductor acknowledges the applause with a deep bow, turns quickly raising their arms. In this moment, the space is pregnant with silence. The conductor is now central to the attention and experience of all, focally positioned between two large groups with different needs but shared agendas. Musicians and audience members alike await a gesture to initiate action or signal the work as underway. The conductor, by virtue of their location and station within the ensemble, is grafted into a “one-way system of communication, running from composer to individual listener through the medium of the performer” (Small, 1998, p. 6) in such a way that they are simultaneously invisible and illuminated to all participants in the musical experience.

The casual observer, and even the experienced musician, might expect to observe synchrony in the moments that follow the conductor’s pregnant pause. Popular culture and mass media create an expectation that musicians synchronize their every gesture with precision and fidelity, coordinating and delivering the musical thoughts of the composer in close coordination with the conductor. One need look no further than the figure of Mickey Mouse in *Fantasia* (Walt Disney Productions and Taylor, 1940) or Leonard Bernstein (1962) to view this paradigm in action: The orchestra’s performance unfurls in synchrony to the time provided by the conductor; their every gesture seems to either directly and immediately illustrate a critical moment of musical activity. These gestures appear to be isochronous to their surrounding temporal environment and ostensibly allow for the coordination of co-performer musical activity (Clayton, 1986; Clayton et al., 2005; Luck and Toiviainen, 2006; Luck and Sloboda, 2007). The conductor’s position in Small’s “one-way system of communication” (1998) appears to fulfill the twin responsibilities outlined by conductor Erich Leinsdorf of “handling traffic and making music” (1981, p. 169). The assumption within that statement being that the conductor is the arbiter of time and affect, and that the timing of the ensemble’s response is in synchrony with the signals conveyed to them.

A cursory examination of practitioner anecdote (Bell, 2004; Johnson, 2014; Todes, 2015; Bennett, 2017) and criticism (Slonimsky, 2000) reveals the presence of greater temporal variability than strict synchrony would admit, most often reported as a delay of ensemble sound to conductor gesture (c.f. Bell, 2004). Corroborating this, career London Symphony Orchestra violist Paul Silverthorne notes that the ensemble plays “behind the beat so they have time to react to the beat, preparing themselves to play properly.” (Todes, 2015, para. 4) Consequently, many conductors anticipate and accommodate an amount of delay, believing that “when an orchestra plays behind the conductor, it has the room to produce a more expressive sound.” (Bennett, 2017, para. 4) Imperfections in a performance’s sonic cohesion are derided as an “intolerable cacophony, an accumulation of strange harmonies that succeed each other without rhythm or sense” (Moreno, in Slonimsky, 2000, p. 197), therefore conductor and ensemble have an expectation to operate in an organized way. The facilitation of organized performance is the explicit focus of numerous conducting texts (Rudolf, 1980; Green and Gibson, 2004; Jordan et al., 2011; Labuta and Matthews, 2018) and consume a significant proportion of conductor training (Manfredo, 2008; Silvey, 2011). The narrow range of temporal variance permitted in evaluations of conductor and ensemble quality (Meals et al., 2019) suggests an outer bound to the aforementioned lag, but the relationship requires a more flexible framing than synchrony provides.

Entrainment, however, offers the degree of temporal variance observed by practitioners. Specific to musical contexts, Philips-Silver and colleagues offer that entrainment is a “spatiotemporal coordination resulting from rhythmic responsiveness” reliant upon “the abilities [of performers] to connect the detection and production of rhythmic information” (2010, p. 7). Informed by the larger human ability to synchronize and adapt to an external, isochronous temporal signals (Large 2000), this ability to coordinate activity with others is fundamental to the creation of music in any group larger than an individual performer. Within music, this phenomenon has been well documented in dyads (Blank and Davidson, 2007; Clayton, 2007; Keller et al., 2007), in small chamber groups (Keller et al., 2014; Timmers et al., 2014; Chang et al., 2017), and in larger music ensembles (D’Ausilio et al., 2012; Volpe et al., 2016; Hilt et al., 2020). Interestingly, the vast majority of research investigating this aspect of musical coordination address the phenomenon at an individual, intraperformer level within those ensembles (D’Ausilio et al., 2012; Glowinski et al., 2015; Hilt et al., 2020), even when additionally assessing the composite performance of the musical performance.

As can be inferred, entrainment exists even within large ensembles, but as a far more complex system. Phillips-Silver et al. (2010) describe music ensembles as representing a special, social instance of entrainment, that is “characterized by a network of input/output connections among individuals in a group (p. 9).” Hilt et al. (2020), exploring intragroup coordination within a chamber orchestra, found channels of sensorimotor communication both within and between performers. Even under experimental manipulation blocking the conductor from the sight of several performers, appropriate musical coordination was maintained. Within this, using Granger (1969), they were able to identify a “clear directionality of the information flow from conductor to musicians” (p. 8) through a leader-follower relationship of bow kinematics to the conductor’s gestures. Using similar methods, D’Ausilio et al. (2012) identify conducted ensemble performance as a “sensorimotor conversation between several individuals” and further posit that, “musicians accommodate their performance according to non-linguistic motor messages received from other musicians and from the conductor” (p. 3).” The role of the conductor in these ensembles is clearly consequential, and the intraensemble communication network that develops to facilitate performance is both robust and complex, but this does not directly speak to the temporal variance noted by performers and practitioners.

The presence of delay in stimulus-response relationships, even in music performance, is not uncommon. The neuroscientific basis of goal-directed behavioral sequences reveals an inherent delay between signal and response, though that delay is seen to decrease with increased activity-specific subject familiarity (Fuster, 1984). In music, the degree of variability observed between internal versus externally mediated timekeeping tasks (Semjen et al., 2000) suggests that these two forces coexist within each individual musician’s performance experience. This adds further support to the findings within investigations of temporal signaling and coordination in music ensembles (D’Ausilio et al., 2012; Glowinski et al., 2015; Hilt et al., 2020). Specifically, the differences reported to focus of attention and interaction in string quartets where the first violinist was asked to surreptitiously vary their interpretation highlight that these channels of communication bear consequence (Glowinski et al., 2015). Glowinski et al. (2015) found that the introduction of a novel interpretation by the nominal quartet leader altered focus of attention, interaction, and self-reported expressivity on the part of other members but left self-reported cohesion largely unaffected. Rather than suggesting that cohesion is invariant in music performance this robustness suggests that it is a foundational component of group music making. Indeed, Keller (2008) reports that individual temporal variations are often sublimated in large groups through the joint action of musicians when the performances are viewed externally. When viewed alongside reported performance lag, this highlights a lacuna in the literature where the interaction of conductors and ensembles is concerned.

While the presence of this conductor-to-ensemble lag is noted in anecdote and supported theoretically, neither the durability of its presence across ensemble types nor its behavior across these contexts or over time are well studied. To this end, the following research questions are proposed:

(1) To what degree does the temporal onset of instrumental ensemble performance vary from the time-bearing information in a conductor’s gesture? Is the variance in this system a static or dynamic feature of ensemble performance?
(2) What are the behavioral characteristics of the temporal variance across common instrumental ensemble configurations? Specifically, ensemble type (wind band and orchestra), experience level (beginner, intermediate, and advanced), rehearsal schedule (beginning, midpoint, and performance), and within selected works.

## Research Design

Audio and video of six instrumental ensembles (three wind band, three orchestra) were recorded in both rehearsal and performance settings. College and secondary ensembles were recruited, consisting of two public junior high schools, one public high school, and one state university (see Table 1). Performers with 1 and 2 years of ensemble-based experience (e.g., junior high school musicians) were considered “Beginner,” while those in high school (3 to 6 years of experience) were considered “Intermediate” and collegiate ensembles (seven or more years of experience) were considered to be “Advanced.” Human subjects permissions were secured from the university and all participant locations, allowing for recruitment of appropriate conductors and ensembles by the researcher. Ensembles were purposefully recruited for consistent artistic excellence as measured through superior contest ratings (Beginner and Intermediate) and critical reception (Advanced), as well as those with conductors in place for two or more years (*M* = 10.8 years, *SD* = 8.3 years). Where a campus supported more than one ensemble, the premier or most advanced group was selected for participation.

**TABLE 1:** Ensemble location and conductor information.

**Ensemble**	**Campus type**	**Grade levels**	**Ensembles per Campus**	**Conductor experience (years)**	**Conductor gender**
Advanced Orchestra	Public University	Undergraduate and Graduate	2	26	Male
Advanced Wind Band	Public University	Undergraduate and Graduate	3	2	Male
Intermediate Orchestra	Public High School	Grade 9–12	3	6	Male
Intermediate Wind Band	Public High School	Grade 9–12	4	9	Female
Beginner Orchestra	Public Junior High School	Grade 6–8	3	9	Female
Beginner Wind Band	Public Junior High School	Grade 6–8	4	13	Female

Appropriate repertoire was identified within each ensemble’s works under preparation and two excerpts were selected per ensemble through the collaboration of researcher and conductor. Selected excerpts fulfilled the following criteria: (1) contrasting tempo and musical material (e.g., slow lyrical and fast active), (2) consistent within-excerpt tempo and clear musical phrase structure, (3) rehearsal preparation had a clear goal orientation (i.e., upcoming performance), (4) no one excerpt began a formal section, movement, or major portion of a given work (see Table 2). Three performance captures per ensemble were evenly divided over 4 weeks, with access dictated by ensemble availability within scheduled rehearsals and school days (*M* = 12 days, *SD* = 6.49 days).

**TABLE 2:** Ensemble repertoire and performance information.

			**Capture 1**		**Capture 2**		**Capture 3**	
			**Excerpt A**	**Excerpt B**	**Excerpt A**	**Excerpt B**	**Excerpt A**	**Excerpt B**
Ensemble	Work Performed	Composer	*Tempo (bpm)*	*Tempo (bpm)*	*Tempo (bpm)*	*Tempo (bpm)*	*Tempo (bpm)*	*Tempo (bpm)*
Advanced Orchestra	*Piano Concerto No. 3 in Bb Minor, Op. 23*	P. I. Tchaikovsky	82	85	80	92	72	92
Advanced Wind Band	*Bells for Stokowski*	M. Daugherty	70	152	88	128	108	164
Intermediate Orchestra	*Serenade, Op. 6*	J. Suk	120	124	118	126	112	120
Intermediate Wind Band	*“March” from Symphonic Metamorphosis on Themes by Carl Maria von Weber*	P. Hindemith	144	144	152	152	148	146
Beginner Orchestra	*Sinfonia in A minor*	G. P. Telemann/arr. B. Mathews	100	98	102	104	98	100
Beginner Wind Band	*The Walking Frog (Two-Step March)*	K. L. King/arr. R. E. Foster	88	90	96	92	100	98

**FIGURE 1:**
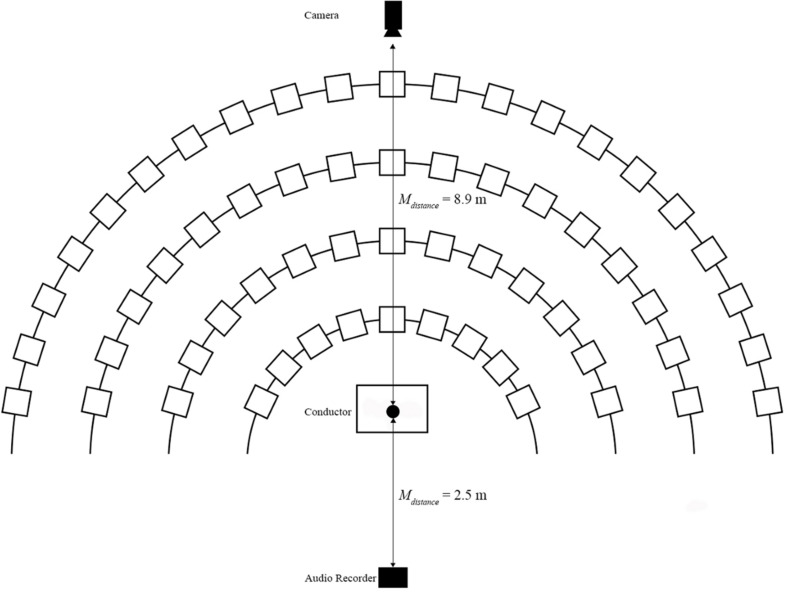
Diagram of ensemble performance capture template. Ensemble performance capture across all ensembles (*N* = 6) was formatted on the above configuration. Slight modifications in conductor-to-camera distance as well as exact placement of camera existed as ensemble setup dictated.

**FIGURE 2:**
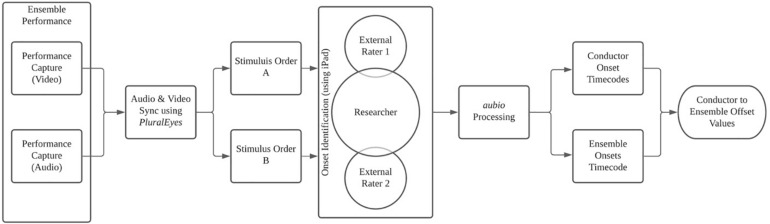
Diagram of audio and video onset processing from capture to offset calculation.

Video was recorded on a Panasonic HC-VX981 4K camcorder (Panasonic, Inc., Japan) placed at the rear of the ensemble facing the conductor (*M*_*distance*_ = 8.9 m). The camera’s viewable field was cropped to highlight only the conductor’s gestures. Audio was simultaneously recorded from the front of the ensemble with a Zoom H6 Handy Recorder (Zoom, North America), placed behind the conductor (*M_*distance*_* = 2.5 m) (see Figure 1). The researcher and conductor conferred to verify the ensemble’s performance quality within each capture to ensure accuracy to the group’s normal efforts. The researcher then synchronized external audio to the performance captured by the camera using PluralEyes (Red Giant, LLC), creating a unified, high-fidelity performance capture. Previously identified excerpts (*M* = 38.52 s, *SD* = 6.42 s) were extracted from this and the resultant stimuli were then separated into audio-only and video-only conditions for onset identification and analysis (see Figure 2). This process was employed to avoid the introduction of novel variables (e.g., MIDI capture, motion capture) into the ensemble’s rehearsal, thereby decreasing the ecological validity of the investigation.

From these, two stimulus orders were prepared by distributing audio-only and video-only stimuli in blocks of like experimental condition but alternating ensemble configuration. Stimuli were ordered in such a way that the audio or video from the same performance appeared at the same point in both orders, but in the opposite context (i.e., audio-only band was followed by audio-only orchestra, with the paired video-only band followed by video-only orchestra in the parallel stimulus order). Each sample was prefixed by a count-down synchronization signal that allowed the researcher to create a meaningful zero point for subsequent onset detection by tapping. Both synchronization and performance onsets were marked by the researcher tapping once per onset (audio or video) with a generic drum sound in GarageBand (Apple, Inc., United States) on an Apple iPad 4. Resultant percussive onsets were exported to mp3 (44.1 kHz) for analysis. To ensure reliability of this measure, a stratified random sample of 10% of onset orders (grouped by ensemble and experience level) was checked by two professional conductors independent of the researcher.

Exported onset audio files of audio-only and video-only stimuli were tagged by condition, ensemble, and capture period and were then processed using the *aubio* audio processing module (Brossier, 2017) in Python 3^1^ using Masri’s high frequency content (Masri, 1996) method with a window size of 1024 (512 bins) to identify onset locations. This method computes frequency-dependent onset locations for an audio signal by “linearly weighting each bin’s contribution in proportion to its frequency” and has been noted for its success with percussion onsets like those used here (Bello et al., 2005). Generated frequency onsets were converted to timecode by dividing the frequency location by the sample rate. Resultant millisecond-scale onsets were matched with their respective audio- and video-only stimuli partners in the opposite stimulus order. Video-only onsets were treated as the basis for comparison to calculate offset quantity between conductor and ensemble, as that conductor-led paradigm conformed to the researcher’s general observations during each capture as well as anecdotal and descriptive accounts on record. This resulted in negative offset values for video-lead/audio-lag conditions and positive values for audio-lead/video-pairings.

Embedded synchronization information (i.e., each stimulus order’s count-down timer) in each sample was used to determine the shared zero-point for each pair. Stimulus orders had been processed three times each by the researcher with acceptable intra-rater reliability using Krippendorff’s Alpha (α = 0.917). Distribution to recruited professional conductors returned a similarly acceptable level of interrater reliability using Krippendorff’s Alpha (α = 0.892)^2^. Generated timecodes of conductor- and ensemble onsets were used to calculate offset quantities which were organized into detailed (grouped by ensemble capture) and mean (averaged at the ensemble level across all captures) orders.

## Analytic Plan

Determining both offset value differences and describing the behavior of these offsets across their appearance creates the need for an analytic plan that allows for a composite examination of the conductor-ensemble relationship. Inferential tools allow for the investigation of differences between ensembles, experience levels, conductors, captures, and conductors, but the qualities of entrainment observed in these ensembles require methods that can adequately describe them. Using methodology described by Clayton et al. (2005), temporal interactions between conductor and ensemble were examined in terms of the relationship between measures of participant latency (via autocorrelation) and patterns in the temporal latency between conductor and ensemble (via relative phase analysis). As the authors note, neither of these modes of analysis by themselves can conclusively analyze entrainment, especially in the absence of emergent performance perturbations, which were structurally omitted from this investigation by the nature of prepared ensemble performance. Given that synchronization and entrainment represent “a complex, dynamic process, not a fixed state” (Clayton et al., 2005) this combination of differences between and behavior within offset values allows for a richer investigation of the relationship between conductor and ensemble than either method in isolation. All analysis was performed in RStudio Team (2020) using R 3.6.1 (R Core Team, 2019).

### Autocorrelation

Autocorrelation generates a series of correlation coefficients for a given variable and a specified number of lagged versions of itself. Here it measures the linear relationship between a given instance of ensemble-to-conductor lag and previous measurements of the same interaction. From this, one can detect whether the values of a variable are dependent on previous values of that same variable, such that the quantity of lag is influenced by preceding lag values. This is presented as an autocorrelation function (ACF) across a given number of lags. Repetitive patterns in these values, represented by the appearance of a “departure and return” to baseline within autocorrelation coefficients, are indicators of underlying relationships within the data series.

### Mean Phase Relationship

Mean phase describes the relationship between two signals in terms of their cyclic occurrence over a sustained period of interaction. In the current study, the latency of the ensemble’s musical performance from the conductor’s perceived temporal information was calculated in a manner described by Clayton et al. (2005). In this, the relationship is expressed as a phase angle (F) calculated by using the previously determined latency of each conductor (C_1_) to ensemble (E_1_) onset factored against the product of all possible phase angles (360) and the lagged interonset interval of the following conductor onset (C_2_) from the onset under investigation. As a formula, this is expressed as F = ((E_1_-C_1_) ^∗^ 360)/(C_2_-C_1_). This process is repeated for all subsequent conductor/ensemble interactions in a given stimuli.

## Results

### Ensemble Differences

The dynamic nature of the interaction between conductor and ensemble across all sampled ensembles and the differences in the behavior of these interactions suggests the potential of quantity differences in temporal lag. All captured excerpts were matched at the beat level and mean values were calculated for all ensembles (see Table 3). Detailed lag values were compared between captures and mean lag values were compared between ensemble type (e.g., wind band, orchestra), conductors, experience levels, conductor gender, and excerpt condition (e.g., fast and slow).

**TABLE 3:** Mean offset values by ensemble.

**Ensemble**	**Mean Offset (ms)**	**Offset SD (ms)**	**Min Offset (ms)**	**Max Offset (ms)**
Beginner Wind Band	25.8	48.6	−195	118
Intermediate Wind Band	1.2	60.2	−125	256
Advanced Wind Band	−113	92.6	−305	18
Beginner Orchestra	−124.8	59.1	−250	−8
Intermediate Orchestra	16.6	51.1	−106	112
Advanced Orchestra	−43.2	112.8	−845	202

*Excerpts were matched across captures at the beat- and phrase level. Resultant beat-level offset values were averaged to calculate mean offsets for each ensemble type.*

**FIGURE 3:**
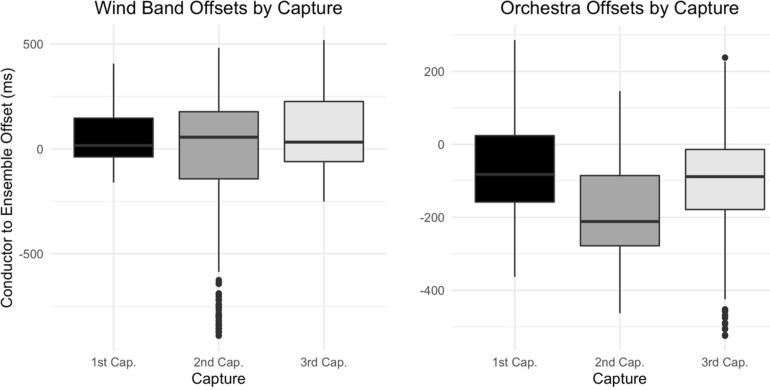
Mean ensemble offsets by capture. Mean offset values by capture where negative values indicate a conductor-led or sight-first relationship.

**FIGURE 4:**
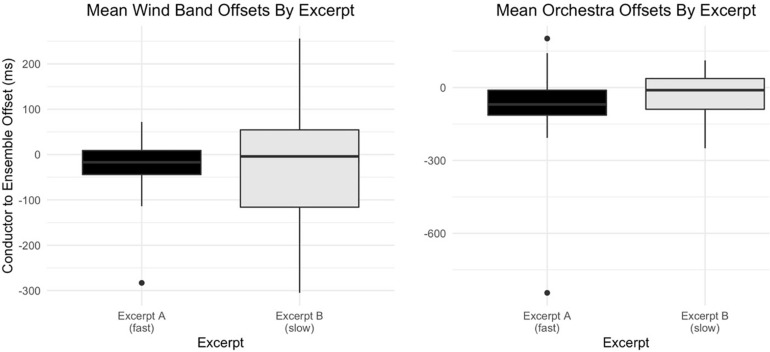
Mean offsets by ensemble and excerpt. Mean offset values by ensemble type and excerpt condition (fast, slow). Negative offset values indicate a conductor-led or sight-first relationship.

An analysis of variance (ANOVA) indicates significant differences by capture [*F*(2,1698) = 62.07, *p* < 0.001, *η_*p*_^2^* = 0.07], though neither ensemble type’s offset values suggest a clear directionality to these differences (see Figure 3). A factorial ANOVA indicates significant differences between mean wind band and orchestra offset values overall [*F*(1,467) = 5.05, *p* < 0.05, *η_*p*_^2^* = 0.01], though a *post hoc* Tukey’s shows no significant differences between excerpt condition within these values (see Figure 4). A significant interaction effect was found between ensemble type and excerpt condition [*F*(1,467) = 8.65, *p* < 0.01, *η_*p*_^2^* = 0.02].

**FIGURE 5:**
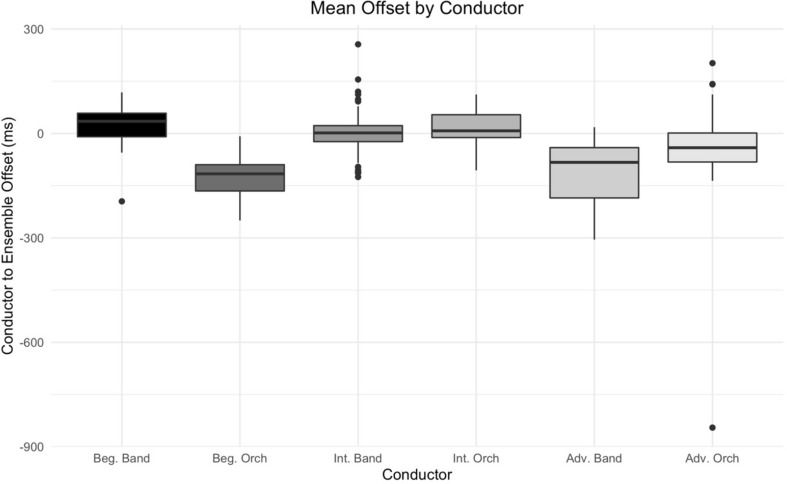
Mean offsets by conductor. Mean offset values by conductor. Significant differences were found between conductors overall, but not between like-ensemble intermediate and advanced conductors.

Additionally, a factorial ANOVA indicates differences in mean offset values between conductors [*F*(5,465) = 61.35, *p* < 0.001, *η_*p*_^2^* = 0.40] using Bonferroni correction for multiple comparisons. A *post hoc* Tukey’s test indicates that differences in offset value exist between some pairings of conductors within a given experience level (*p* < 0.01) but not between like-ensemble conductors across experience levels; specifically finding a lack of significant differences within pairs of advanced and intermediate band and orchestra conductors, respectively (see Figure 5). Overall, however, a factorial ANOVA indicates significant differences by in mean offset value by ensemble experience level [*F*(2,468) = 41.35, *p* < 0.001, *η_*p*_^2^* = 0.15].

No significant differences were found between excerpt type (fast, slow) or between conductor gender.

### Autocorrelation

**FIGURE 6:**
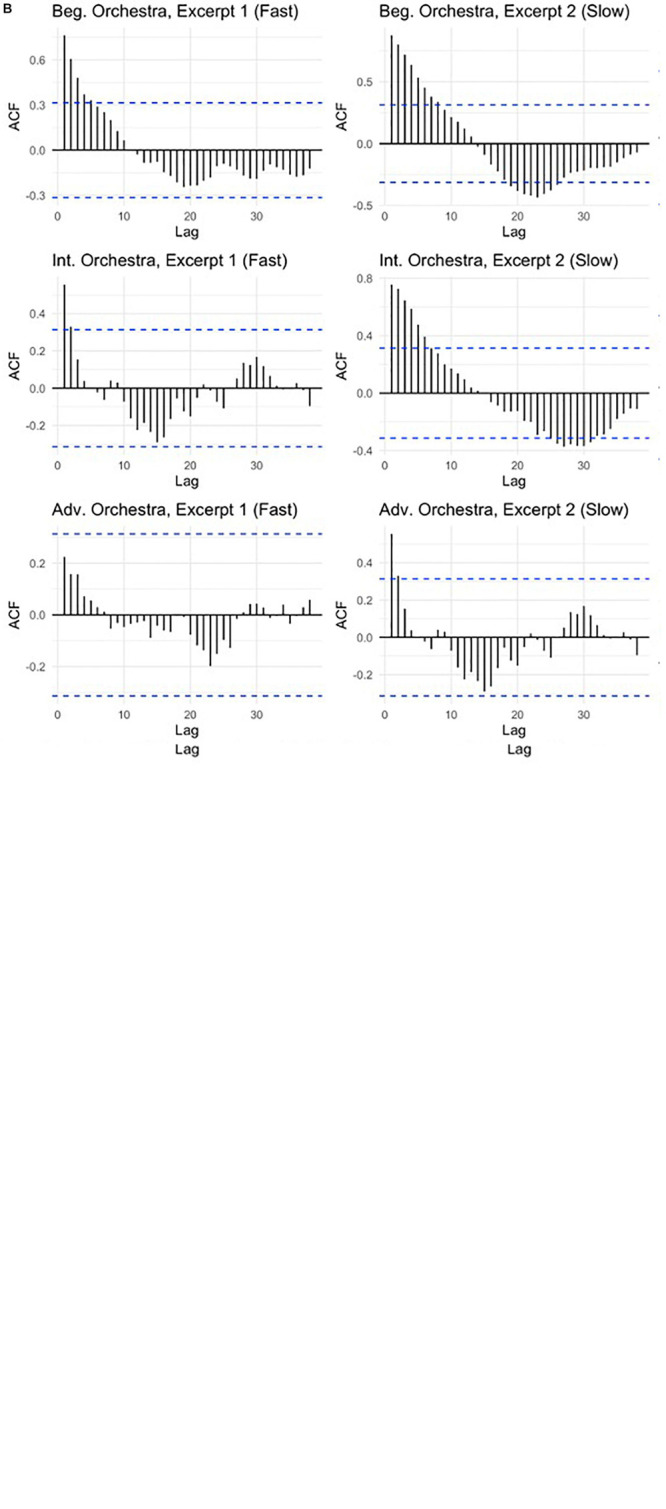
**(A)** Wind band autocorrelations of mean time series by excerpt and experience. **(B)** Orchestra autocorrelations of mean times series by excerpt and experience. Autocorrelation function (ACF) values indicate the correlation of mean conductor-ensemble offset instances with lagged values across a time series. A cyclical departure-and-return pattern can be seen in many examples, indicating that the quantities of offset correlate over time. This suggests a progression or seasonality to changes in those values over the course of a given excerpt.

All time series met the criteria for dependence as measured by the Ljung–Box test save for Intermediate Band Excerpt 2, Advanced Band Excerpt 1, and Advanced Orchestra Excerpt 1. Dependence here suggests that there is significance to the pattern of serial autocorrelations within the time series, indicating an identifiable pattern to its changes. Examination of the conductor-to-ensemble lag value autocorrelations reveals an expected “departure and return” dynamic, though differences appear to exist between fast and slow excerpts (see Figures 6A,B). The contrast of tighter side-lobe groupings in fast excerpts to the longer-term patterns in slow excerpts is clearer at the intermediate and advanced levels, most notably within the Intermediate Orchestra and Advanced Band performances.

### Mean Phase Relationship

Computed phase relationships help to highlight the degree of temporal difference between conductor and ensemble where 0° indicates complete synchrony or phase lock and ±180° indicates perfect asynchrony or a complete anti-phase relationship with positive values indicating the temporal primacy of conductor onsets, and negative values indicating an ensemble-led relationship. While phase values for orchestra (*M* = 26.8°, *SD* = 53.5°) and wind band (*M* = 24.3°, *SD* = 71.4°) were not found to be significantly different, the behavior of these phase relationships indicates potential differences between ensemble type and experience level.

The general tendency of Beginner and Intermediate Wind Bands to move between conductor- and ensemble-led behavior, clustering near synchrony (0°), contrasts with the tendency of Beginner and Advanced Orchestra, as well as Advanced Wind Band, which generally demonstrate conductor-led performance. Additionally interesting is the similarity between Intermediate Wind Band and Orchestra, both tending toward an ensemble-led phase relationship, especially in their slow excerpts. As each ensemble’s excerpts were matched between captures at the phrase level this most strongly bears consideration where the ensemble’s repertoire is concerned, though the orientation of these ensembles to consequential competitive performance likely also bears further investigation.

**FIGURE 7:**
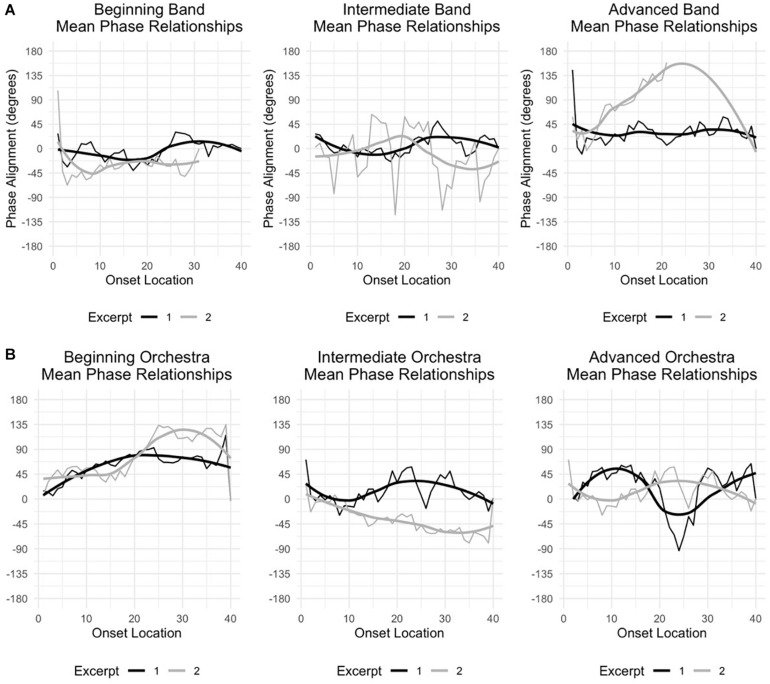
**(A)**: Mean wind phase relationships by experience and excerpt. **(B)**: Mean orchestra phase relationships by experience and excerpt. Phase relationships illustrate the behavior of offset values over their appearance, here by ensemble and excerpt. Negative phase values indicate a sound-first or ensemble led relationship, while positive phase values indicate a sight-first or conductor-led relationship. A general tendency for wind band to vary between conductor- and ensemble-led relationships can be contrasted against a general tendency of orchestra toward a conductor-led relationship. Exceptions [e.g., Intermediate Orchestra, Excerpt 2 (Slow), Advanced Band, Excerpt 2 (Slow)] may indicate features of the ensemble’s preparation rather than generalizable features of the ensemble’s behavior.

The leader-follower dynamic was generally observed in all ensembles with notable differences in the quality of the interaction between ensemble types (see Figures 7A,B). Overall, orchestras generally demonstrated a conductor-led interaction across all experience levels where wind bands demonstrated a more complicated leader-follower dynamic. Especially in Excerpt 1 (fast), one can see the propensity of the wind band to anticipate the gesture of the conductor, in essence serving as the temporal leader. Similarly, the vast majority of wind band slow excerpt observations showcase a propensity toward both ensemble anticipation, and the conductor-led relationship broadly seen in the orchestras.

## Discussion

The presence of a perceptible lag between conductor gesture and ensemble response is anecdotally present (Bell, 2004; Johnson, 2014; Todes, 2015; Bennett, 2017), but empirical investigation of its features and behavior are largely absent the existing scholarship. This exploratory study suggests that observed offset is not a static property of ensemble function but is in fact a flexible property of internal temporal interaction that indicates a dynamic relationship between conductor and ensemble. While a precise description of the intraensemble relationship falls outside the scope of this paper, some noteworthy features do emerge.

The offsets observed within sampled ensembles generally support anecdotal accounts of the conductor-as-leader relationship implied by terms like “orchestral lag” (Bell, 2004; Bennett, 2017). Mean offset values for wind band (*M* = -31.1 ms, *SD* = 93 ms) and orchestra (*M* = -50.8 ms, *SD* = 98.2 ms), where negative values indicate a sight before sound (here, conductor before ensemble) relationship, suggest an a general tendency toward conductor-led interactions in orchestra but ensemble-led interactions in wind bands. The effect sizes in these findings were generally small, however, suggesting that additional consideration is required.

Differences in ensembles highlighted by offset quantity are further supported by the difference in offset behavior seen through the comparison of phase relationships between ensemble types (see Figures 7A,B). In this, wind band phase values are roughly distributed between a conductor- and ensemble-lead where orchestral phase values indicate a general tendency toward a conductor-led relationship. Notable exceptions exist within both ensemble types (e.g., Intermediate Orchestra and Advanced Band), but the broad trend is otherwise consistent. This further confirms long-standing anecdote and conforms to experiences informally shared by a collaborating conductor whose experience encompasses both ensemble contexts, noting that “The [wind] band just has more immediacy to it, [it’s] a more impatient thing. The orchestra takes its time … it waits to see what you’re going to do and then decides to go along with you … or not.” (personal communication, February 2018).

Differences across experience levels and captures also indicate differences in degree of offset but fail to conclusively demonstrate differences in kind. In other words, the absolute differences in offset value quantity and behavior found between secondary (Beginner, Intermediate) and tertiary (Advanced) ensembles suggest the possibility that a performer’s experience in these ensembles plays a role in these differences, but the current data do not present a clear linear relationship. Additionally, differences found between ensemble captures indicate changes over short-term ensemble development, but the study was not designed to reveal if these changes possess a robust linear relationship in any ensemble or ensemble type. A stabilization of phase relationship, as seen through reduced phase variability in the third wind band capture, may represent the stable-but-flexible, mature relationship of musical actors noted by Clayton (2012) and others (Phillips-Silver et al., 2010; Levitin et al., 2018), though further research is required in this specific setting.

Of interest within these many differences and similarities, however, are the behavior of phase relationships within Beginner and Intermediate wind bands. The presence of an asymmetric entrainment relationship (Clayton, 2012), in which the conductor’s motion is the only time-bearing signal coordinated with, is assumed to an almost foundational level in ensembles of this type (Rudolf, 1980; Leinsdorf, 1982; Green and Gibson, 2004; Jordan et al., 2011). Interestingly, these two ensembles regularly make use of an audible metronome during rehearsal. Though they refrained during this study’s performance captures, the behavior of phase relationships in these ensembles reveal a wide absolute variance that centers around synchrony, moving between conductor-led and ensemble-led orientations both within and between captures. This behavior suggests the potential for another temporal signal within the performance, imposing an additional signal that performers are attempting to entrain to. Though it is speculative, this could be evidence of the aforementioned metronome engendering a form of self-entrainment on the part of individual musicians or the group as a whole (Phillips-Silver et al., 2010).

These findings are limited in several ways that bear discussion and consideration in the final interpretation of the results. The broad nature of the audio data analyzed (group onset) does not allow for the consideration of temporally consequential aspects of wind and string instrument performance, where the differences in frequency propagation and response time between woodwind, brass, string, and percussion instruments present a wide range of affordances to be considered in future investigations (Benade, 1969; Rossing, 2010). Additionally, the sample size of recruited ensembles (*N* = 6) coupled with the significance of the findings and size of effects between ensembles, experience levels, and chronological development demonstrates the need for further research in this area. In addition, intergroup differences could illustrate a conductor-effect, where an individual’s pedagogy and gesture interact with performers in a unique manner. The large effect size found in comparisons of offsets by conductor supports this potential and Alan Gilbert, former music director of the New York Philharmonic, further leavens this possibility in his statement that, “there is a connection between the gesture, the physical presence, the aura that a conductor can project, and what the musicians produce” (Gilbert, 2012).

For many outside of ensemble music, the complex and dynamic ecosystem inside these groups appears to be dominated and guided by the interaction of sound and the conductor’s baton. Great amounts of responsibility and power have been ascribed to the individual atop the podium, but an emerging body both of practice and research calls into question the singularity of this individual’s role in the coordination of the ensemble’s musical efforts. The conductor’s role as a source of entrainment continues to be clear, but the findings of this study offer support for the growing body of research indicating the influence of other time-bearing actors within the ensemble, even if only by describing the outlines of their effects.

The differences described here – between ensemble types, experience levels, and chronological growth – are present in all groups and time periods sampled, though to differing degrees. These differences – both in degree of lag and in its behavior revealed through relative phase and autocorrelation – present a fruitful avenue for future investigation where the mechanics of each ensemble’s instrumentation combine with both conductor-to-musician intra-ensemble and musician-to-musician inter-performer communication. An increase in our understanding of this dense web of entrainment and interaction that describe communication and coordination in ensembles will have numerous benefits to our understanding of group music performance and allow for the refinement of a comprehensive, nuanced, and accurate model of ensemble performance. This, in turn, has the potential to support both the performer and educator pedagogy and practice by allowing for a more granular and realistic understanding of the volume and tenor of activity across an network where they represent only one node but where their actions can have a global impact.

## Data Availability Statement

The raw data supporting the conclusions of this article will be made available by the authors, without undue reservation.

## Ethics Statement

The studies involving human participants were reviewed and approved by the Research, Integrity, and Oversight (RIO) Office University of Houston, Division of Research 1-713-743-9204 cphs@central.uh.edu. Written informed consent from the participants’ legal guardian/next of kin was not required to participate in this study in accordance with the National Legislation and the Institutional Requirements.

## Author Contributions

The author confirms being the sole contributor of this work and has approved it for publication.

## Conflict of Interest

The author declares that the research was conducted in the absence of any commercial or financial relationships that could be construed as a potential conflict of interest.

## Footnotes

1 http://www.python.org

2 Krippendorff’s Alpha, a test of inter-rater reliability, is utilized here for its robust reliability across multiple coders and the non-binary manner in which it deals with disagreement between (*m*) coders Krippendorff (2012). The data being evaluated (individual onsets, *SD* = 183 ms) combined with computationally generated factor weights and a conservative lower bound (α = 0.800) all contribute to its use in this investigation.
