# Supplementary material for: The Question of Lag: An Exploration of the Relationship Between Conductor Gesture and Sonic Response in Instrumental Ensembles
Source: Front Psychol. 2020 Dec 10;11:573030. doi: 10.3389/fpsyg.2020.573030 (PMC7758255; doi:10.3389/fpsyg.2020.573030)
Supplement: Supplementary file 3 [file Data_Sheet_3.PDF]

Frontiers in Psychology: Manuscript 573030

Data File Variable Descriptions

Data Sheet 1 Description

Variable Name	Variable Type	Value Range	Description
Capture	*Categorical*	1..3	Ensemble performance capture
Excerpt	*Categorical*	1..2	Excerpt captured (e.g. Fast, Slow)
Ensemble	*Categorical*	1..2	Ensemble type (e.g., Band, Orchestra)
XP	*Categorical*	1..3	Ensemble experience level (e.g., Beginner, Intermediate, Advanced)
Offset	*Integer*	-890.93 .. 581.57	Value (in ms) of conductor-to-ensemble response offset

Data Sheet 2 Description

Variable Name	Variable Type	Value Range	Description
Offset.Loc	*Categorical*	1..40	Offset location in excerpt
Excerpt	*Categorical*	1..2	Excerpt captured (e.g. Fast, Slow)
Ensemble	*Categorical*	1..2	Ensemble type (e.g., Band, Orchestra)
XP	*Categorical*	1..3	Ensemble experience level (e.g., Beginner, Intermediate, Advanced)
Gender	*Categorical*	1..2	Conductor gender (e.g., Male, Female)
Cond	*Categorical*	1..6	Conductor ID
Offset	*Integer*	-845 .. 256	Mean value for offset location across all captures
Phase	*Integer*	-122.07° .. 397.13°	Phase relationship between conductor and ensemble
