# Supplementary material for: The Question of Lag: An Exploration of the Relationship Between Conductor Gesture and Sonic Response in Instrumental Ensembles
Source: Front Psychol. 2020 Dec 10;11:573030. doi: 10.3389/fpsyg.2020.573030 (PMC7758255; doi:10.3389/fpsyg.2020.573030)
Supplement: Supplementary file 4 [file Data_Sheet_4.PDF]

Frontiers in Psychology: Manuscript 573030

R Scripts

Chunk 1: Packages and Descriptives

```
knitr::opts_chunk$set(echo = FALSE)
LagAllDF <- readr::read_csv("")
DetOffset <- readr::read_csv("")
library(tidyverse)
library(stats)
library(magrittr)
library(TTR)
library(forecast)
library(grid)
library(gridExtra)
library(Rmisc)


head(LagAllDF)
head(DetOffset)


LagAllDF$Excerpt <- as.factor(LagAllDF$Excerpt)
LagAllDF$Ensemble <- as.factor(LagAllDF$Ensemble)
LagAllDF$Level <- as.factor(LagAllDF$Level)
LagAllDF$Gender <- as.factor(LagAllDF$Gender)
LagAllDF$Conductor <- as.factor(LagAllDF$Conductor)

DetOffset$Capture <- as.factor(DetOffset$Capture)
DetOffset$Ensemble <- as.factor(DetOffset$Ensemble)

LagAllDF %>%
  group_by(Ensemble) %>%
  dplyr::summarize(mean=mean(Phase, na.rm=TRUE),
                   sd = sd(Phase, na.rm=TRUE),
                   min = min(Phase, na.rm=TRUE),
                   max = max(Phase, na.rm=TRUE))

BandData <- LagAllDF %>%
  filter(Ensemble==1)
head(BandData)
OrchData <- LagAllDF %>%
```

```
  filter(Ensemble==2)
head(OrchData)
```

Chunk 2: Inferrential Statistics & Hypothesis Testing

```
t.test(LagAllDF$Offset~LagAllDF$Ensemble)
t.test(LagAllDF$Offset~LagAllDF$Excerpt)
t.test(LagAllDF$Offset~LagAllDF$Gender)

ModelCap <- lm(Offset.MS ~ Capture*Ensemble,
                data=DetOffset)
summary(ModelCap)
Cap.stdres <- rstandard(ModelCap)
qqnorm(Cap.stdres)
qqline(Cap.stdres)
ModelLevel <- lm(Offset ~ Level*Ensemble,
                  data=LagAllDF)
summary(ModelLevel)
Lev.stdres <- rstandard(ModelLevel)
qqnorm(Lev.stdres)
qqline(Lev.stdres)
ModelCond <- lm(Offset ~ Conductor*Ensemble,
                data=LagAllDF)
summary(ModelCond)
Cond.stdres <- rstandard(ModelCond)
qqnorm(Cond.stdres)
qqline(Cond.stdres)

DetEnsAOV <- aov(Offset ~ Ensemble * Level * Conductor, data=LagAllDF)
   summary(DetEnsAOV, p.adjust="bonferroni")
   TukeyHSD(DetEnsAOV, p.adjust="bonferroni")
CapAOV <- aov(Offset.MS ~ Capture * Ensemble, data=DetOffset)
   summary(CapAOV, adjust="bonferroni")
   TukeyHSD(CapAOV, adjust="bonferroni")
LevelAOV <- aov(Offset ~ Level, data=LagAllDF
   summary(LevelAOV, adjust="bonferroni")
   TukeyHSD(LevelAOV, adjust="bonferroni")
CondAOV <- aov(Offset ~ Conductor, data=LagAllDF)
   summary(CondAOV,adjust="bonferroni")
   TukeyHSD(CondAOV, adjust="bonferroni")
```

Chunk 3: Visualization - Offsets by Excerpt, Experience, Conductor

```
Band.Excerpt <- factor(BandData$Excerpt,labels = c("Excerpt A\n(fast)","Excerpt
B\n(slow)"))
```

```
Orch.Excerpt <- factor(OrchData$Excerpt,labels = c("Excerpt A\n(fast)","Excerpt
B\n(slow)"))
a <- ggplot(data=BandData, aes(x=Band.Excerpt, y=Offset, group=Band.Excerpt,
fill=Band.Excerpt)) +
    geom_boxplot() +
    ggtitle("Mean Wind Band Offsets By Excerpt") +
        xlab("Excerpt") +
        ylab("Conductor to Ensemble Offset (ms)") +
        labs(fill = "Excerpt") +
    theme_minimal() +
    theme(legend.position = "none", plot.title=element_text(hjust=0.5))
a <- a + scale_fill_grey(start = 0, end = .9)
b <- ggplot(data=OrchData, aes(x=Orch.Excerpt, y=Offset, group=Orch.Excerpt,
fill=Orch.Excerpt)) +
    geom_boxplot() +
    ggtitle("Mean Orchestra Offsets By Excerpt") +
        xlab("Excerpt") +
        ylab("   ") +
        labs(fill = "Excerpt") +
    theme_minimal() +
    theme(legend.position="none", plot.title=element_text(hjust=0.5))
b <- b + scale_fill_grey(start = 0, end = .9)


Band.XP <- factor(BandData$Level, labels = c("Beg.","Int.","Adv."))
Orch.XP <- factor(OrchData$Level, labels = c("Beg.","Int.","Adv."))
WBXP <- ggplot(data=BandData, aes(x=Band.XP, y=Offset, fill=Band.XP,
group=Band.XP)) +
    geom_boxplot() +
    ggtitle("Mean Wind Band Offsets by\nExperience Level") +
        xlab("Experience Level") +
        ylab("Conductor to Ensemble Offset (ms)") +
    theme_minimal() +
    theme(legend.position="none", plot.title=element_text(hjust=0.5))
WBXP <- WBXP + scale_fill_grey(start = 0, end = .9)
ORXP <- ggplot(data=OrchData, aes(x=Orch.XP, y=Offset, fill=Orch.XP,
group=Orch.XP)) +
    geom_boxplot() +
    ggtitle("Mean Orchestra Offsets by\nExperience Level") +
          ylab("   ") +
          xlab("Experience Level") +
    theme_minimal() +
    theme(legend.position="none", plot.title=element_text(hjust=0.5))
ORXP <- ORXP + scale_fill_grey(start = 0, end = .9)
```

```
png("/Users/corymeals/Desktop/MeanOffsetsByEnsXP.png", width = 9, height = 4,
units = 'in', res = 300)
multiplot(WBXP,ORXP, cols=2)
png("/Users/corymeals/Desktop/MeanOffsetsByExcerpt.png", width = 9, height = 4,
units = 'in', res = 300)
multiplot(a,b, cols = 2)
dev.off()

Cond.ID <- factor(LagAllDF$Conductor, labels = c("Beg. Band", "Beg. Orch", "Int.
Band", "Int. Orch", "Adv. Band", "Adv. Orch"))

COND.DET <- ggplot(LagAllDF, aes(x=Cond.ID, y=Offset, group=Cond.ID)) +
  geom_boxplot(aes(fill = Cond.ID)) +
  ggtitle("Mean Offset by Conductor") +
    ylab("Conductor to Ensemble Offset (ms)") +
    xlab("Conductor") +
  theme_minimal() +
   theme(legend.position="none", plot.title=element_text(hjust=0.5))
COND.DET <- COND.DET + scale_fill_grey(start = 0, end = .9)
```

Chunk 4: Visualization - Offsets by Capture

```
Band.Plot <- DetOffset %>% filter(Ensemble==1)
    Band.Cap <- factor(Band.Plot$Capture, labels = c("1st Cap.","2nd Cap.","3rd
Cap."))
Orch.Plot <- DetOffset %>% filter(Ensemble==2)
    Orch.Cap <- factor(Orch.Plot$Capture, labels = c("1st Cap.","2nd Cap.","3rd
Cap."))
WBBP <- ggplot(data=Band.Plot, aes(x=Band.Cap, y=Offset.MS, fill=Band.Cap,
group=Band.Cap)) +
    geom_boxplot() +
    ggtitle("Wind Band Offsets by Capture") +
        xlab("Capture") +
        ylab("Conductor to Ensemble Offset (ms)") +
         labs(fill = "Capture") +
    theme_minimal() +
    theme(legend.position="none", plot.title=element_text(hjust=0.5))
WBBP <- WBBP + scale_fill_grey(start = 0, end = .9)
ORBP <- ggplot(data=Orch.Plot, aes(x=Orch.Cap, y=Offset.MS, fill=Orch.Cap,
group=Orch.Cap)) +
    geom_boxplot() +
    ggtitle("Orchestra Offsets by Capture") +
          ylab("   ") +
          xlab("Capture") +
    theme_minimal() +
```

```
    theme(legend.position="none", plot.title=element_text(hjust=0.5))
ORBP <- ORBP + scale_fill_grey(start = 0, end = .9)
```

Chunk 5: Visualization - Mean Phase Relationships

```
PhaseDF_Beginner <- LagAllDF %>% filter(Level==1)
BPhaseDF_Excerpt <- factor(PhaseDF_Beginner$Excerpt, labels(c("Excerpt 1","Excerpt
2")))
PhaseDF_BBand <- PhaseDF_Beginner %>% filter(Ensemble==1)
BBPhaseDF_Excerpt <- factor(PhaseDF_BBand$Excerpt, labels(c("Excerpt 1","Excerpt
2")))
PhaseDF_BOrch <- PhaseDF_Beginner %>% filter(Ensemble==2)
BOPhaseDF_Excerpt <- factor(PhaseDF_BOrch$Excerpt, labels(c("Excerpt 1","Excerpt
2")))

PhaseDF_Int <- LagAllDF %>% filter(Level==2)
PhaseDF_IBand <- PhaseDF_Int %>% filter(Ensemble==1)
IBPhaseDF_Excerpt <- factor(PhaseDF_IBand$Excerpt, labels(c("Excerpt 1","Excerpt
2")))
PhaseDF_IOrch <- PhaseDF_Int %>% filter(Ensemble==2)
IOPhaseDF_Excerpt <- factor(PhaseDF_IOrch$Excerpt, labels(c("Excerpt 1","Excerpt
2")))

PhaseDF_Adv <- LagAllDF %>% filter(Level==3)
PhaseDF_ABand <- PhaseDF_Adv %>% filter(Ensemble==1)
ABPhaseDF_Excerpt <- factor(PhaseDF_ABand$Excerpt, labels(c("Excerpt 1","Excerpt
2")))
PhaseDF_AOrch <- PhaseDF_Adv %>% filter(Ensemble==2)
AOPhaseDF_Excerpt <- factor(PhaseDF_AOrch$Excerpt, labels(c("Excerpt 1","Excerpt
2")))

BB <- ggplot(data=PhaseDF_BBand,
       aes(x=Offset.Loc, y=Phase, color=BBPhaseDF_Excerpt,
group=BBPhaseDF_Excerpt)) +
       geom_line()+
        geom_smooth(aes(color=BBPhaseDF_Excerpt), method="loess", se=F,
inherit.aes = TRUE) +

 scale_y_continuous(limits=c(-180,180),breaks=c(-180,-135,-90,-45,0,45,90,135,180)
) +
        ggtitle("Beginning Band\nMean Phase Relationships") +
        xlab("Onset Location") +
        ylab("Phase Alignment (degrees)") +
         labs(color="Excerpt") +
    theme_minimal() +
```

```
    theme(legend.position="bottom", plot.title=element_text(hjust=0.5))
BB <- BB + scale_color_grey(start = 0, end = .7)

BO <- ggplot(data=PhaseDF_BOrch,
       aes(x=Offset.Loc, y=Phase, color=BOPhaseDF_Excerpt,
group=BOPhaseDF_Excerpt)) +
       geom_line()+
        geom_smooth(aes(color=BOPhaseDF_Excerpt), method="loess", se=F,
inherit.aes = TRUE) +

 scale_y_continuous(limits=c(-180,180),breaks=c(-180,-135,-90,-45,0,45,90,135,180)
) +
       ggtitle("Beginning Orchestra\nMean Phase Relationships") +
        xlab("Onset Location") +
        ylab("  ") +
        labs(color="Excerpt") +
     theme_minimal() +
    theme(legend.position="bottom", plot.title=element_text(hjust=0.5))
BO <- BO + scale_color_grey(start = 0, end = .7)

IB <- ggplot(data=PhaseDF_IBand,
       aes(x=Offset.Loc, y=Phase, color=IBPhaseDF_Excerpt)) +
       geom_line()+
        geom_smooth(aes(color=IBPhaseDF_Excerpt), method="loess", se=F,
inherit.aes = TRUE) +

 scale_y_continuous(limits=c(-180,180),breaks=c(-180,-135,-90,-45,0,45,90,135,180)
) +
        ggtitle("Intermediate Band\nMean Phase Relationships") +
        xlab("Onset Location") +
        ylab("Phase Alignment (degrees)") +
        labs(color="Excerpt") +
     theme_minimal() +
    theme(legend.position="bottom", plot.title=element_text(hjust=0.5))
IB <- IB + scale_color_grey(start = 0, end = .7)

IO <- ggplot(data=PhaseDF_IOrch,
       aes(x=Offset.Loc, y=Phase, color=IOPhaseDF_Excerpt)) +
       geom_line()+
        geom_smooth(aes(color=IOPhaseDF_Excerpt), method="loess", se=F,
inherit.aes = TRUE) +

 scale_y_continuous(limits=c(-180,180),breaks=c(-180,-135,-90,-45,0,45,90,135,180)
) +
       ggtitle("Intermediate Orchestra\nMean Phase Relationships") +
        xlab("Onset Location") +
```

```
        ylab("  ") +
        labs(color="Excerpt") +
     theme_minimal() +
    theme(legend.position="bottom", plot.title=element_text(hjust=0.5))
IO <- IO + scale_color_grey(start = 0, end = .7)

AB <- ggplot(data=PhaseDF_ABand,
       aes(x=Offset.Loc, y=Phase, color=ABPhaseDF_Excerpt)) +
       geom_line()+
        geom_smooth(aes(color=ABPhaseDF_Excerpt), method="loess", se=F,
inherit.aes = TRUE) +

 scale_y_continuous(limits=c(-180,180),breaks=c(-180,-135,-90,-45,0,45,90,135,180)
) +
        ggtitle("Advanced Band\nMean Phase Relationships") +
        xlab("Onset Location") +
        ylab("Phase Alignment (degrees)") +
        labs(color="Excerpt") +
    theme_minimal() +
    theme(legend.position="bottom", plot.title=element_text(hjust=0.5))
AB <- AB + scale_color_grey(start = 0, end = .7)

AO <- ggplot(data=PhaseDF_AOrch,
       aes(x=Offset.Loc, y=Phase, color=AOPhaseDF_Excerpt)) +
       geom_line()+
        geom_smooth(aes(color=AOPhaseDF_Excerpt), method="loess", se=F,
inherit.aes = TRUE) +

 scale_y_continuous(limits=c(-180,180),breaks=c(-180,-135,-90,-45,0,45,90,135,180)
) +
       ggtitle("Advanced Orchestra\nMean Phase Relationships") +
        xlab("Onset Location") +
        ylab("  ") +
        labs(color="Excerpt") +
    theme_minimal() +
    theme(legend.position="bottom", plot.title=element_text(hjust=0.5))
AO <- AO + scale_color_grey(start = 0, end = .7)
```

Chunk 6: Visualization - Time Series (ACF)

```
BB.1 <- filter(LagAllDF, Level == 1, Ensemble == 1, Excerpt == 1)
BB.2 <- filter(LagAllDF, Level == 1, Ensemble == 1, Excerpt == 2)
BO.1 <- filter(LagAllDF, Level == 1, Ensemble == 2, Excerpt == 1)
BO.2 <- filter(LagAllDF, Level == 1, Ensemble == 2, Excerpt == 2)
IB.1 <- filter(LagAllDF, Level == 2, Ensemble == 1, Excerpt == 1)
```

```
IB.2 <- filter(LagAllDF, Level == 2, Ensemble == 1, Excerpt == 2)
IO.1 <- filter(LagAllDF, Level == 2, Ensemble == 2, Excerpt == 1)
IO.2 <- filter(LagAllDF, Level == 2, Ensemble == 2, Excerpt == 2)
AB.1 <- filter(LagAllDF, Level == 3, Ensemble == 1, Excerpt == 1)
AB.2 <- filter(LagAllDF, Level == 3, Ensemble == 1, Excerpt == 2)
AO.1 <- filter(LagAllDF, Level == 3, Ensemble == 2, Excerpt == 1)
AO.2 <- filter(LagAllDF, Level == 3, Ensemble == 2, Excerpt == 2)

BB.1.TS <- ts(BB.1$Offset, start = 1, end = 39)
BB.2.TS <- ts(BB.2$Offset, start = 1, end = 39)
BO.1.TS <- ts(BO.1$Offset, start = 1, end = 39)
BO.2.TS <- ts(BO.2$Offset, start = 1, end = 39)
IB.1.TS <- ts(IB.1$Offset, start = 1, end = 39)
IB.2.TS <- ts(IB.2$Offset, start = 1, end = 39)
IO.1.TS <- ts(IO.1$Offset, start = 1, end = 39)
IO.2.TS <- ts(IO.2$Offset, start = 1, end = 39)
AB.1.TS <- ts(AB.1$Offset, start = 1, end = 39)
AB.2.TS <- ts(AB.2$Offset, start = 1, end = 39)
AO.1.TS <- ts(AO.1$Offset, start = 1, end = 39)
AO.2.TS <- ts(AO.2$Offset, start = 1, end = 39)

A1 <- ggAcf(BB.1.TS, lag.max=39) +
  ggtitle("Beg. Wind Band, Excerpt 1 (Fast)") +
        xlab("Lag") +
        ylab("ACF value") +
        theme_minimal()

A2 <- ggAcf(BB.2.TS, lag.max=39) +
  ggtitle("Beg. Wind Band, Excerpt 2 (Slow)") +
        xlab("Lag") +
        ylab("ACF") +
        theme_minimal()
A3 <- ggAcf(BO.1.TS, lag.max=39) +
  ggtitle("Beg. Orchestra, Excerpt 1 (Fast)") +
        xlab("Lag") +
        ylab("ACF") +
        theme_minimal()
A4 <- ggAcf(BO.2.TS, lag.max=39) +
  ggtitle("Beg. Orchestra, Excerpt 2 (Slow)") +
        xlab("Lag") +
        ylab("ACF") +
        theme_minimal()
A5 <- ggAcf(IB.1.TS, lag.max=39) +
  ggtitle("Int. Wind Band, Excerpt 1 (Fast)") +
        xlab("Lag") +
        ylab("ACF") +
```

```
        theme_minimal()
A6 <- ggAcf(IB.2.TS, lag.max=39) +
  ggtitle("Int. Wind Band, Excerpt 2 (Slow)") +
        xlab("Lag") +
        ylab("ACF") +
        theme_minimal()
A7 <- ggAcf(IO.1.TS, lag.max=39) +
  ggtitle("Int. Orchestra, Excerpt 1 (Fast)") +
        xlab("Lag") +
        ylab("ACF") +
        theme_minimal()
A8 <- ggAcf(IO.2.TS, lag.max=39) +
  ggtitle("Int. Orchestra, Excerpt 2 (Slow)") +
        xlab("Lag") +
        ylab("ACF") +
        theme_minimal()
A9 <- ggAcf(AB.1.TS, lag.max=39) +
  ggtitle("Adv. Wind Band, Excerpt 1 (Fast)") +
        xlab("Lag") +
        ylab("ACF") +
        theme_minimal()
A10 <- ggAcf(AB.2.TS, lag.max=39) +
  ggtitle("Adv. Wind Band, Excerpt 2 (Slow)") +
        xlab("Lag") +
        ylab("ACF") +
        theme_minimal()
A11 <- ggAcf(AO.1.TS, lag.max=39) +
  ggtitle("Adv. Orchestra, Excerpt 1 (Fast)") +
        xlab("Lag") +
        ylab("ACF") +
        theme_minimal()
A12 <- ggAcf(AO.2.TS, lag.max=39) +
  ggtitle("Adv. Orchestra, Excerpt 2 (Slow)") +
        xlab("Lag") +
        ylab("ACF") +
        theme_minimal()
```
